# Identifier mapping performance for integrating transcriptomics and proteomics experimental results

**DOI:** 10.1186/1471-2105-12-213

**Published:** 2011-05-27

**Authors:** Roger S Day, Kevin K McDade, Uma R Chandran, Alex Lisovich, Thomas P Conrads, Brian L Hood, VS Kumar Kolli, David Kirchner, Traci Litzi, G Larry Maxwell

**Affiliations:** 1 Department of Biomedical Informatics, University of Pittsburgh School of Medicine, Pittsburgh, PA 15261 USA; 2 Department of Biostatistics, University of Pittsburgh, Pittsburgh, PA 15260 USA; 3 Women's Health Integrated Research Center at Inova Health System, Inova Fairfax Hospital Campus, 3300 Gallows Road, Falls Church, VA 22042 USA; 4 Windber Research Institute, 620 Seventh Street, Windber, PA 15963 USA; 5 Division of Gynecologic Oncology, Walter Reed Army Medical Center, Washington, D.C. 20307 USA

## Abstract

**Background:**

Studies integrating transcriptomic data with proteomic data can illuminate the proteome more clearly than either separately. Integromic studies can deepen understanding of the dynamic complex regulatory relationship between the transcriptome and the proteome. Integrating these data dictates a reliable mapping between the identifier nomenclature resultant from the two high-throughput platforms. However, this kind of analysis is well known to be hampered by lack of standardization of identifier nomenclature among proteins, genes, and microarray probe sets. Therefore data integration may also play a role in critiquing the fallible gene identifications that both platforms emit.

**Results:**

We compared three freely available internet-based identifier mapping resources for mapping UniProt accessions (ACCs) to Affymetrix probesets identifications (IDs): DAVID, EnVision, and NetAffx. Liquid chromatography-tandem mass spectrometry analyses of 91 endometrial cancer and 7 noncancer samples generated 11,879 distinct ACCs. For each ACC, we compared the retrieval sets of probeset IDs from each mapping resource. We confirmed a high level of discrepancy among the mapping resources. On the same samples, mRNA expression was available. Therefore, to evaluate the quality of each ACC-to-probeset match, we calculated proteome-transcriptome correlations, and compared the resources presuming that better mapping of identifiers should generate a higher proportion of mapped pairs with strong inter-platform correlations. A mixture model for the correlations fitted well and supported regression analysis, providing a window into the performance of the mapping resources. The resources have added and dropped matches over two years, but their overall performance has not changed.

**Conclusions:**

The methods presented here serve to achieve concrete context-specific insight, to support well-informed decisions in choosing an ID mapping strategy for "omic" data merging.

## Background

Regulation of protein abundance is a central determinant of cellular phenotype. Therefore the ability to conduct and interpret studies of proteome-wide alterations in protein abundance presents tremendous promise for biological understanding. Proteomics based on MS/MS (tandem mass spectrometry) enables direct detections of peptide fragments for identification and quantitation of proteins in a proteome-wide manner. However, it has some major handicaps, especially detection biases and low dynamic range[1] (though techniques requiring labeling can have great dynamic range[2]). Hybridization-based expression microarrays represent a well-established high-throughput technology for conducting global measurements of mRNA transcript abundances. However, although mRNA expression precedes protein translation, the correlation between transcript level and abundance of the corresponding protein product, is often poor [3].

Thus neither transcriptomic nor proteomic studies are perfect. However, when performed on the same samples they are complementary [4,5]. A relevant analogy comes from statistics. Central to many statistical procedures (such as empirical Bayes estimation) is the established principle that combining two data sources with different sources of bias and variance frequently produces greater precision than either alone[6,7]. Genomic and proteomic data sets have different sources of bias and variance, so combining them may lead to a more precise view of differential protein abundance. Consider one application, biomarker discovery. Improving the selection of candidates to validate is a worthy goal, since biomarker validation is generally elaborate and costly. If both transcriptomic and proteomic platforms agree on a strong differential expression between the groups of patients to be distinguished, the attractiveness of a candidate strengthens. If not, the call is for caution.

The potential contributors to poor correlations are numerous. Post-transcriptional events such as alternative splicing and microRNA regulation complicate the link between the abundance of a specific mRNA and production of its protein product. Thus microarray transcript signals may not faithfully reflect the pool of transcripts available for translation. On the other hand, proteins which degrade quickly will be underrepresented compared to those with greater half-lives[8], so variation in protein degradation can also reduce the correlation between transcriptomics and proteomics. In summary, decoupled expression at the mRNA and protein levels might relate to post-transcription and post-translation events; explanations might be forthcoming from studies of microRNA-mediated regulation and protein degradation [4,9].

But the decoupling might not be biological; it might stem from errors in the data integration. The supposed identity of either the gene coding for the probeset's target transcript or the detected protein may be incorrect. The quality of a study integrating proteomic and genomic data rests heavily on reliable mapping between the identifiers of the two high-throughput platforms. Discrepancies between bioinformatics identifier mapping resources are abundant. Draghici [10] has demonstrated a variety of serious ID mapping anomalies, in which results depend strikingly on which bioinformatics mapping resource is chosen. Identifier assignments for genes, mRNA species, and proteins are managed and the annotations curated by different bioinformatics centers. The annotation systems of the microarray probesets depend on the chip manufacturer to provide mappings to transcript identifiers, so the provenance or motivation behind a link between an array probeset and a mRNA species may be unclear. In addition, in mass spectrometry the incidence of misidentification of protein accessions is not negligible, for a host of reasons, including variations in sequence search algorithms and tuning parameters. Therefore for consumers of these resources, to determine with accuracy how a protein connects with a mRNA species and thence to an microarray probeset can be labor-intensive and error-prone[11].

The possibility of misidentifications is troubling in biomarker discovery projects. When a candidate marker appears promising, misidentification of the protein or transcript will lead to wasted effort in initial marker validation and/or subsequent clinical prediction studies. With two integrated discovery platforms, on the other hand, if a candidate appears promising in one of the platforms but ID mapping generates a poor correlation with the other platform, then the integration is useful by casting suspicion on the reported identity of the biomarker.

Despite the appeal of integrated genomic and proteomic analysis, it is rarely done, and more rarely still is the identifier mapping methodology described. But there are pioneering examples of integrated studies. Chen [5] studied gene expression using the Plus 2.0 Affymetrix chip together with proteomic analysis using Isotope-Coded Affinity Tags (ICAT) methodology. The focus was high-risk neuroblastoma, with a small number of clinical stage 4 samples with MYCN amplified and stage 1's with MYCN not amplified. It was unclear how identifier mapping was performed. Another study performed by Shankavaram et al.[4] used a protein lysate array and the Affymetrix U133 Plus 2.0 chip to identify biomarkers present on the NCI-60 cancer cell panel data. Matchminer[12] was used to obtain annotation matches. Chen et al. 2002 [13] used integrated analysis on 76 lung adenocarcinoma and nine non-neoplastic lung tissues. The report mentions that an elevation in protein did not always correlate with an elevated mRNA expression level. The identifier mapping resource is not mentioned for this study.

The motivation for our study of this issue was a transcriptome-proteome integrated study comparing early endometrial cancer with normal endometrial tissue from cancer-free subjects[14]. Preliminary efforts quickly revealed major anomalies in some of the identifier matches. This result motivated a deeper investigation into the fidelity of identifier mapping, to achieve acceptable reliability of the linkages between the two data sets. The starting point is the proteomic study, generating UniProt ACCs. With these ACCs we queried three prominent bioinformatics identifier mapping resources, to obtain corresponding Affymetrix probeset identifiers. There will be a multitude of mapping strategies obtainable by connecting combinations of bioinformatics resources, but these three are the only ones that we are aware of providing a direct mapping query suitable for this specific purpose. Inconsistencies encountered here are likely emblematic of all mapping strategies. Here we report the extent of agreements and disagreements among the resources' returned results.

We also utilized the two datasets to generate correlations between message expression and protein expression. Such correlations have been studied before. Yu et al [9] provide a discussion of the kinetics of the expected relation between mRNA expression and protein expression, together with supplemental data that suggests that correlation values may vary according to GO-defined functional grouping, including some groups with negative correlations. Nie et al [15] studied mRNA-protein correlations in *Desulfovibrio vulgaris*. They found that mRNA abundance explains 20-28% of protein abundance variation, and functional category explains 10-15% of variation in correlation. We decomposed the correlations into a mixture with a zero-centered component and a positive component. The mixture distribution model sheds light on the degree to which positive correlations exceed negative ones, allowing estimation of the distribution of correlations among correctly mapped protein-probeset pairs, without needing to know at this point which specific pairs are correctly mapped and which are not. The only assumption is that the distribution of correlations among the mismatched pairs is symmetric around zero. We proceeded presuming that better identifier matching should generate a higher rate of match pairs with strong correlations between the transcript signals and corresponding protein spectral counts. This is flawed as a "gold standard" because of issues discussed above: pre- and post-translation events which decouple expression at the mRNA and protein levels[4,9]. Nevertheless, large observed correlations are likely to be more prevalent when the ID mapping is done correctly, and less likely when done incorrectly. We demonstrate that the ensemble of correlations is useful for evaluating mapping services even though any individual correlation is not.

This paper first presents summaries of the retrieval sets from each individual resource, then presents comparisons between pairs of resources, and finally evaluates the mappings based on the assay correlations. The overall objective is to develop and demonstrate methods that bring a needed critical but constructive eye to integrative studies.

## Results

### Samples and data

We obtained fresh frozen endometrial cancer tissue specimens from 91 stage I endometrial patients and seven age-matched normal endometrial samples from post-menopausal women. Proteomic spectral count data analyzed was the sum of four LC-MS/MS analyses from two laboratories. The combined analyses yielded 11,879 distinct protein UniProt ACCs across all samples and both instruments. Transcript analysis using the Affymetrix U133 Plus 2.0 chip provided gene expression data. Details are in the Methods section.

### Annotation systems

We restricted the focus to bioinformatics resources providing direct mappings between UniProt ACCs and Affymetrix probeset IDs. Identifier mapping systems examined included the Affymetrix NetAffx Analysis Center[16]; ENFIN's EnVision and Ensembl resources[17]; and the DAVID resource (Database for Annotation, Visualization, and Integrated Discovery by NIAID[18,19]. Details of the annotation systems and our methods for accessing them are provided in the Methods section at the end of this paper. Submitting the list of 11,879 UniProt ACCs to each resource provided putative matches to probesets on the Affymetrix U133 Plus 2.0 chip. We refer to the mapping retrievals obtained interactively from NetAffx as **NetAffx_Q **or **Aff_Q**. We label results from processing downloaded data files as **NetAffx_F or Aff_F**. We refer to results from the EnVision query web services as **EnVision_Q **or **EnV_Q**. We refer to results obtained programmatically from the EnSembl GUI as **EnSembl_F **or **EnS_F**. The labels **DAVID_Q or D_Q **will refer to results obtained from the DAVID web service application programming interface (API). In addition we obtained DAVID Knowledgebase files by request from the NIAID. We label probeset match retrievals from DAVID Knowledgebase files as **DAVID_F or D_F**. In general the suffix "Q" refers to methods using a direct query method, whether programmatic or interactive, while "F" refers to methods using downloaded files.

Our results concentrate primarily on the "Q" query-based retrievals; similarities and contrasts with the "F" file-based retrievals are noted as appropriate. To evaluate performance changes over time we also present earlier results, obtained variously in 2008 and 2009, labeling the earlier results as above but with the suffix "**_8**".

### Distribution of the number of probesets retrieved by each resource

Beginning with the UniProt ACCs returned by Sequest[20] for our proteomic tandem mass spectrometry experiment, we characterized each annotation resource individually by the distribution of the number of corresponding probeset IDs returned for each ACC. Table 1 shows the percentage of ACCs with 0, 1, 2, 3 or more probesets found.

**Table 1:** Distribution of the number of probesets retrieved for each UniProt ACC, by bioinformatics resource.

	# Affymetrix probesets returned						
		
	0	1	2	3	>3	max #probesets returned	total # probesets returned
		
*2010*							
DAVID_Q	*15%*	*27%*	*21%*	*14%*	*22%*	34	28077
EnVision_Q	*26%*	*38%*	*21%*	*9%*	*5%*	11	15650
NetAffx Q	*18%*	*31%*	*23%*	*13%*	*15%*	48	23441
***2008-9***							
**D Q_8**	*19%*	*31%*	*22%*	*13%*	*15%*	18	22608
**EnV_Q_8**	*23%*	*41%*	*23%*	*9%*	*4%*	15	15916
**Aff Q_8**	*29%*	*32%*	*19%*	*10%*	*10%*	53	19256

The proportion of ACCs returning at least one corresponding probeset ID was 84.7% for DAVID_Q, 73.6% for EnVision_Q, and 72% for Affy_Q. EnVision_Q returned the fewest probesets, and was most likely to return only a single match (38.2% versus 27.1% for DAVID_Q and 31.2% for Affy_Q).

**Figure 1:**
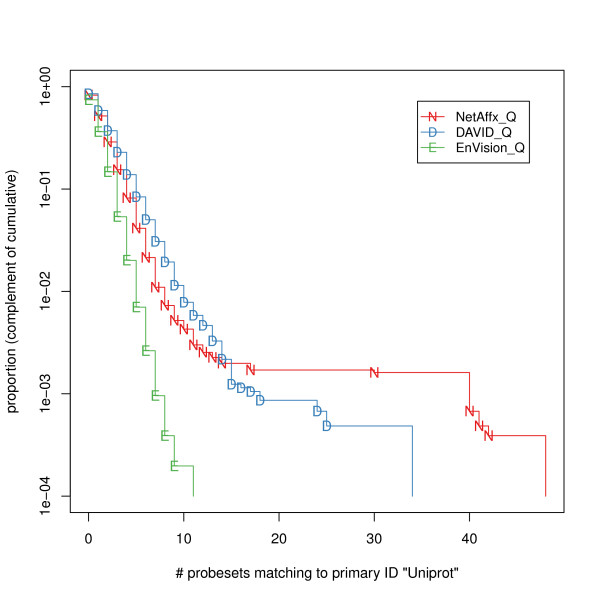
**Distribution of the number of probesets retrieved for each UniProt ACC**. Complementary empirical cumulative distribution functions, plotted on a vertical log scale to emphasize differences.

Figure 1 shows the behavior of the large-cardinality retrieved match sets. EnVision_Q delivers fewer matches overall, and also a lower proportion of intermediate-sized and large sets. NetAffx_Q delivers a few large sets. For example, for one Uniprot ACC, A2NYU9, NetAffx_Q returned 40 probe sets while DAVID_Q and EnVision_Q returned only one probe set. The reasons for these discrepancies may be diverse. From NetAffx_Q there were 81 large (40 or larger) match sets, associated with ten immunoglubulin heavy chain genes and one gene coding for a zinc finger protein.

### Pairwise comparisons of identifier mapping resources

**Figure 2:**
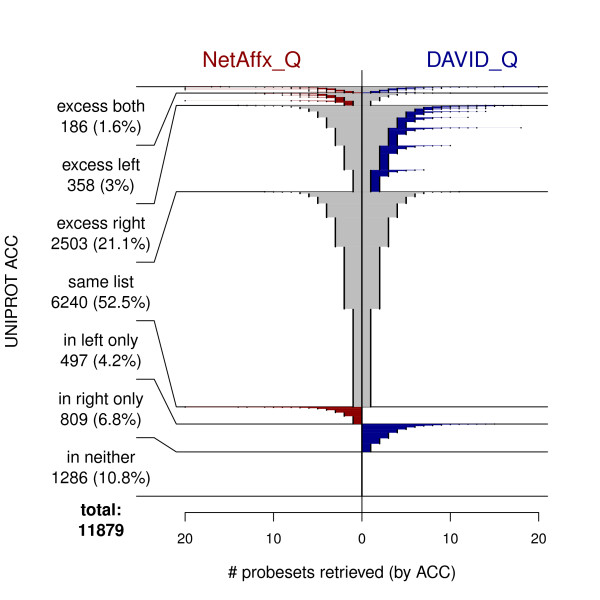
**Fountain plot comparing retrievals from NetAffx_Q and DAVID_Q**. **Horizontal axis**: For each UniProt ACC, the number of probesets retrieved from NetAffx _Q (left of zero) and DAVID _Q (right of zero). **Vertical axis**: Each horizontal slice is one ACC (11,879 slices are stacked). ACCs were categorized, and the probeset counts for each ACC graphed by category, sorted within category by intersection size, then by total size. **Category definition example**: "excess left" is the set of ACCs such that both probeset retrievals were non-empty, and {probesets retrieved by NetAffx _Q} is a strict subset of {probesets retrieved by DAVID_Q}.

We compared each pair of annotation resources by constructing for each UniProt ACC the intersection and set differences between the two probeset lists mapping to that ACC. The results across all ACCs were grouped according to whether there were no matches in either resource, no matches in one resource with one or more matches in the other, two identical non-empty match sets, one match set but not the other reporting extra matches (containing the other match set), or extra matches reported by each resource. The fountain plot of Figure 2 compares NetAffx_Q and DAVID_Q in this way. The ACC counts and proportions appear at the left of the figure. The figure is constructed by stacking 11,879 horizontal lines; each horizontal line is one ACC. It therefore shows the classification and probeset retrieval size, for each of the 11,879 UniProt ACCs, by category. Within category the results are sorted vertically by the size of the intersection, followed by the sum of the two retrieval set sizes (length of each horizontal line).

**Figure 3:**
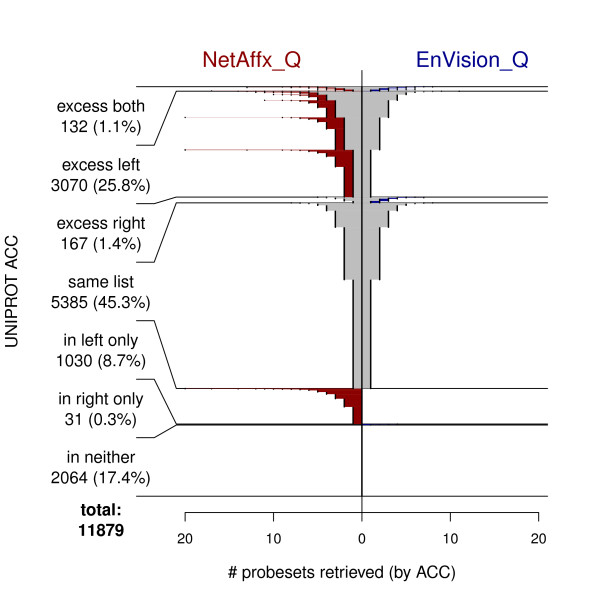
**Fountain plot comparing web interfaces: NetAffx_Q vs EnVision_Q**.

**Figure 4:**
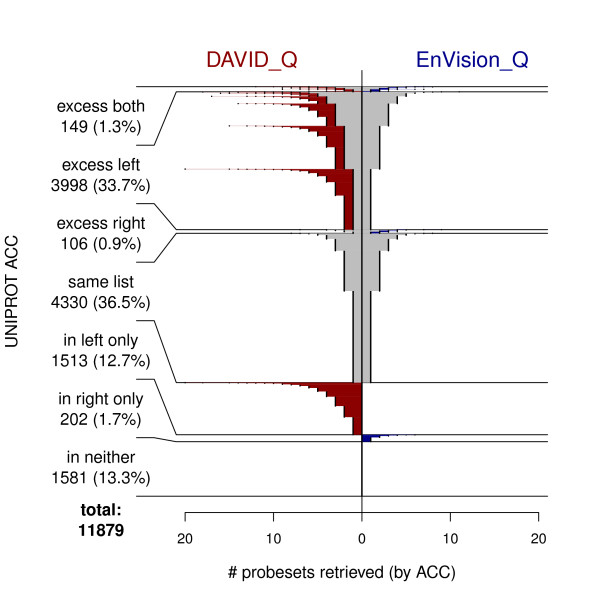
**Fountain plot comparing web interfaces: DAVID_Q vs EnVision_Q**.

The extent of the disagreements among resources is not insignificant. NetAffx_Q and DAVID_Q returned identical non-empty answers for 52.5% of the ACCs (For 10.8%, no probesets were found by either resource.). Figures 3 and 4 show less agreement between EnVision_Q and either NetAffx_Q or DAVID_Q; less than half of the ACCs. returned identical answers. Furthermore, each application processed a substantial number of ACCs by returning 1, 2, and occasionally many more probesets when the other two resources produced no matches. This result is exemplified by the horizontal extent of the red and blue portions of the lines in Figures 2, 3, and 4.

In the past two years, the proportion of ACCs matched to identical non-empty lists has increased somewhat for NetAffx and DAVID (from 39% to 52%) and for NetAffx and EnVision (from 31% to 45%), but decreased slightly for DAVID and EnVision (from 39% to 36.5%). For NetAffx, the number of ACCs with at least one match not previously present is 1899, and the number of ACCs previously present but no longer matching is 504; the proportion of ACCs with identical non-empty NetAffx lists between 2008 and 2010 is 46% (5529/11879).

**Figure 5:**
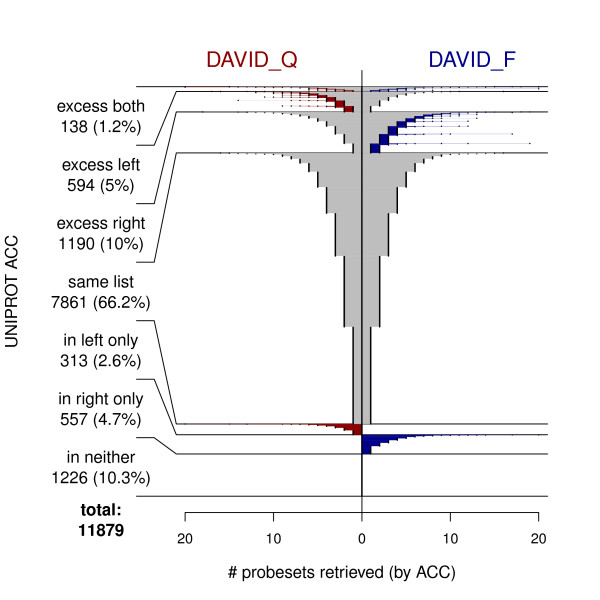
**Fountain plot comparing DAVID file-based to query-based ID mappings**.

Contrasts between online query and file download services were of interest. Comparing DAVID_Q with DAVID_F (Figure 5), there are respectively 2.6% and 4.6% excess matches, as well as 5% and 9.9% additional (in excess of up to 19) matches. In contrast, NetAffx Q and F yield exactly the same match sets; however, this perfect agreement may be a quirk of timing; previously we have found them to differ. EnV_Q and EnS_F differed by only two UniProt ACCs.

From personal correspondence with the NetAffx and DAVID resource teams we learned that the tradeoffs between the speed and timeliness of access vs. reliability differ considerably between the resources. From the developers of DAVID, it follows that the files available on request are more accurate and more current than the DAVID web query service. On the other hand, obtaining the DAVID file data at the moment cannot be fully automated, since it requires sending a request to the DAVID team and a wait for the provision of a temporary download URL. Choosing the online query to the DAVID database over the file download would be preferable when the wait is not acceptable. In contrast, access to the Affymetrix files is instant. However, from discussions with an Affymetrix representative, we learned that the results of a NetAffx query gradually evolve with limited curation between releases of the Affymetrix annotation file, which is fully manually curated and released roughly quarterly. Software tools provided by Affymetrix generally use the annotation files, not live web queries. Due to the timing of our recent accesses, the two recent retrievals were identical.

### Mapping all probesets to ACCs

So far our results utilize ACCs provided by Sequest database in analysis of a particular set of 98 samples, as inputs into mapping resources, representing an archetypal use case for ID mapping. Reversing the direction of the mapping, one can also utilize all of the probeset IDs on a microarray as inputs, to characterize all ACCs that would be mapped, independent of any particular proteomic experiment. With the U133 Plus 2.0 array, the total numbers of ACCs retrieved are seen in Table 2. DAVID_F returns ACCs for the human ALU probeset **affx-hum_alu_at **(5165). The other occasions of very high counts are probesets that map to MHC genes.

**Table 2:** UniProt ACCs retrieved by mapping all probesets on U133 Plus 2.0

	**# ACCs retrieved**						
		
	**0**	**1**	**2**	**3**	**>3**	**max # ACCs returned**	**total # ACCs returned**
		
***2010***							
**DAVID_F**	*15%*	*8%*	*11%*	*13%*	*54%*	5165	293199
**DAVID_Q**	*16%*	*4%*	*8%*	*12%*	*60%*	1657	303145
**EnVision_Q**	*41%*	*14%*	*12%*	*10%*	*23%*	2388	140699
**Affy_Q**	*26%*	*14%*	*13%*	*11%*	*36%*	2185	210944

The large discrepancy for DAVID_Q appears related to its higher conformity to SwissProt; 74% of the ACCs returned by DAVID_Q are in SwissProt, versus 23.5% for Aff_Q and 25.4% for EnVision_Q. In comparison, of the 11879 ACCs originally returned by Sequest for the MS/MS experiment, 80.0% are in SwissProt. Among those, the subsets mapping to at least one probeset match by DAVID_Q, Aff_Q and EnV_Q are all primarily in SwissProt (89.7%, 86.3%, and 92.% respectively). Thus, the three resources are much more similar on a "real-world" set of ACCs from an experiment than one would expect from the comprehensive probeset-to-ACC maps of Tables 3 and 4.

**Table 3:** Total numbers of ACCs returned for the U133 Plus 2.0 array.

*Union*	*Intersection*	*Aff_Q*	*EnV_Q*	*D_Q*	*D_Q only*	*not in D_Q*
140752	37989	81306	73247	97063	54283	30095
(100%)	( 27%)	( 58%)	( 52%)	( 69%)	( 39%)	( 21%)

Of 140752 ACCs returned for U133 plus 2.0 by any of DAVID_Q, EnVision_Q or Affy_Q ("*Union*"), only 37989 were returned by all three ("*Intersection*"). A large proportion (39%) were returned only by DAVID.

**Table 4:** For each service, the collection of probesets with at least one mapped ACC.

*Service returns at least one mapped ACC*			#Probesets			
***Affy_Q***	***DAVID_Q***	***EnV_Q***	**Any ACC**		**At least one SwissProt**	

No	No	No	7256	(13%)	8499	(16%)
✓	No	No	898	(2%)	533	(1%)
No	✓	No	6593	(12%)	6881	(13%)
✓	✓	No	7710	(14%)	8940	(16%)
No	No	✓	174	(0%)	134	(0%)
✓	No	✓	444	(1%)	340	(1%)
No	✓	✓	433	(1%)	352	(1%)
✓	✓	✓	31167	(57%)	28996	(53%)

**TOTAL**			54675	(100%)	54675	(100%)

Counts are for each "Venn diagram" subset of services indicated in columns 1-3. For example, 13% of probesets return no ACC from any service; 16% return no Swissprots. Only 57% of probesets have at least one ACC returned by all three services. As was seen in the "0" column of Table 2, DAVID returns answers for 85% of probesets, 74% for NetAffx, and 59% for EnVision. 80% of ACCs are SwissProt ACCs.

### Annexin 2: Example of variation of transcriptome-proteome correlations for individual proteins

To study mappings for individual proteins, we utilized the MS/MS and U133 Plus 2.0 microarray data sets described above. For each match of an ACC to a probeset ID, we merged the corresponding subsets of MS/MS and microarray data by subject ID.

We consider one protein that appears to be elevated in abundance in endometrial cancer relative to normal tissue, annexin 2 (UniProt ACC = P07355). Retrievals are shown in Table 5.

**Table 5:** Probeset retrievals for annexin A2, and Spearman correlations with annexin A2 spectral counts.

*Probeset*	*1568126_at*	*201590_x_at*	*210427_x_at*	*213503_x_at*	*210876_at*	*211241_at*
**DAVID Q**	✓	✓	✓	✓	✓	✓

**EnVision_Q**		✓	✓	✓		

**NetAffx Q**	✓	✓	✓	✓		

**Correlation w/spectral count**	0.176	0.532	0.531	0.557	0.321	0.305

**Pseudogene**^**1**^					✓	✓

**Annotation Grade**^**1**^	E	A	A	A	A	A

**Exonic good match**^**2**^	0	4	4	3	11	6

**Exonic poor match**^**2**^	11	1	1	1	0	4

**Intronic**^**2**^	0	0	0	0	0	1

**Exonic Cross Hybridized**^**2**^	0	6	6	7	0	0

^1 ^From NetAffx web site ^**2 **^From Plandbaffy [21]

**Figure 6:**
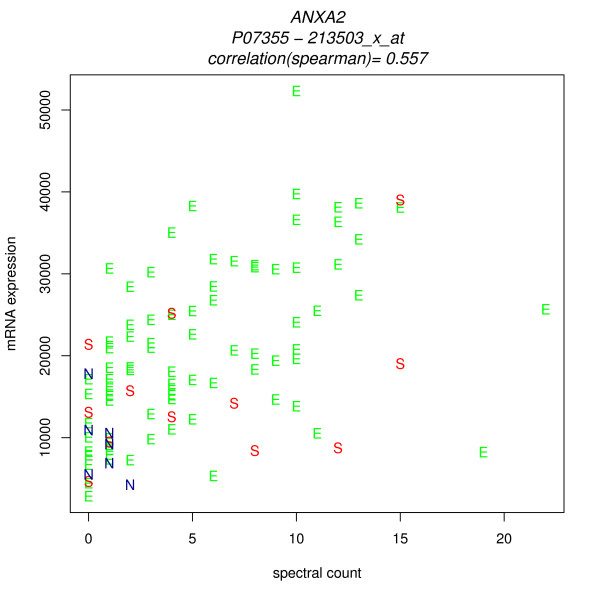
**Scatterplot, 213503_x_at transcript signals versus Annexin 2 spectral counts**, E = endometrioid cancer, S = serous cancer, N = normal.

**Figure 7:**
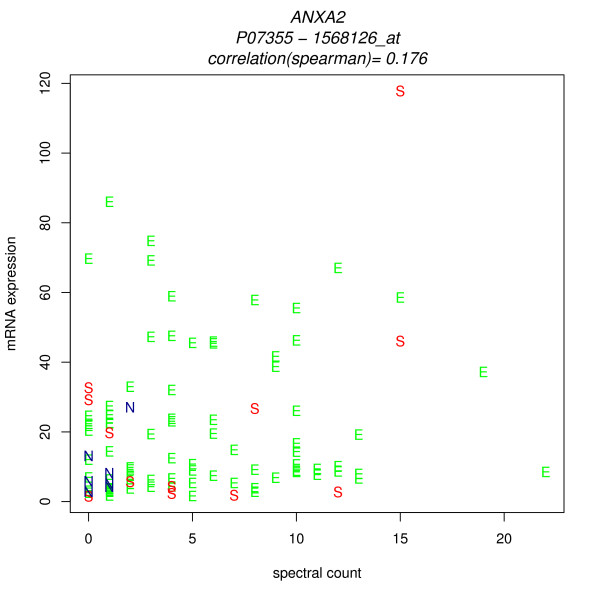
**Scatterplot, 1568126 _at transcript signals versus Annexin 2 spectral counts**, E = endometrioid cancer, S = serous cancer, N = normal.

Figures 6 and 7 show merged data scatterplots for the two probesets with the best and the worst correlation. (The other probesets are strongly correlated with the ANXA2 spectral counts, and with 213503_x_at. One match, 211241_at, is new; it was not a match in DAVID_Q_8, EnV_8 or NetAffx_8. It has moderate correlations with the other probesets except 1568126_at. ) The presence of strong correlations between protein spectral counts and most of the probesets reinforces confidence in the correct identification of the protein, and in the validity of the cancer-associated differential expression.

The presence of one poor correlation does not lessen that confidence. This example highlights the fact that a poor correlation does not necessarily mean that the mRNA and protein levels are truly biologically decoupled. The cause of the poor correlation may be an incorrect mapping or a probe-specific assay anomaly. In the case of 1568126_at, further investigation yields an explanation: the NetAffx annotation grade for 1568126_at is "E", indicating mapping only to an expressed sequence tag. Note that the probeset ID "_x_at" quality tag, which indicates caution because some probes hit transcripts from different genes, does not provide guidance; in fact it corresponds to the best correlations. (Affymetrix documentation affirms that the ID assignment at the time of array design is necessarily permanent and reflects the limited knowledge at that time). Analysis of the sequences of the probeset target and individual probes is warranted, and under way, but beyond the scope of this study.

### Evaluation of mapping correctness by correlation analysis

To assess the quality of the identifier matches, we performed merges as described in the previous section for every UniProt-probeset pair obtained from one of the mapping strategies. Each corresponding pair of protein spectral counts with microarray expression signals yielded a Spearman correlation. The rationale for examining the entire collection of correlations is as follows. High correlations would be likely, though not guaranteed, to indicate a correct ID match. Negative correlations or correlations plausibly generated by chance might indicate any of several possibilities: (a) the ID match could be incorrect; (b) any of several biological phenomena could cause message expression to fail to manifest proportionately in protein abundance; and/or (c) measurement error variance and bias could mask a true biological correlation. With these limitations in mind, the collection of correlations was used to evaluate the performance of each system to generate correct matches. This analysis includes only the 480 ACCs with at least an average of 0.5 MS/MS spectral events per sample.

**Figure 8:**
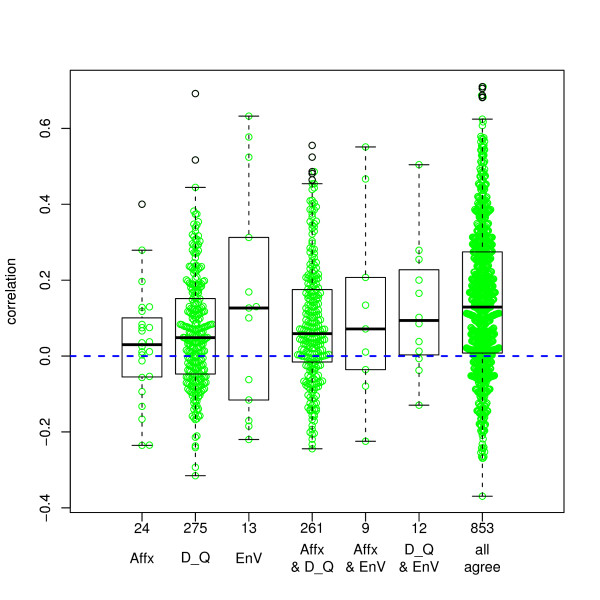
**Correlation distributions by match group**. Correlations between log(mRNA) levels and spectral counts. Box extends to the first and third quartile, with thick horizontal line at the median. The group "all" constitutes matches that all three resources returned (i.e., the intersection); "Affx only" constitutes matches returned only by NetAffx Q; "EnV&D_Q" constitutes matches returned by EnVision_Q and DAVID Q but not by NetAffx_Q; etcetera.

**Figure 9:**
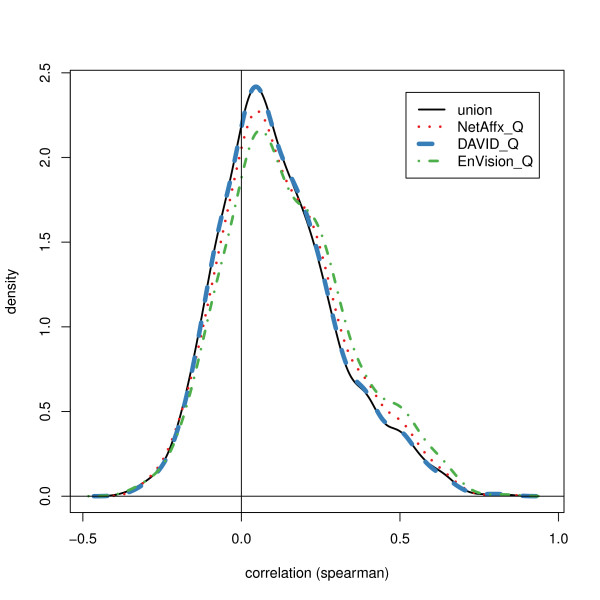
**Estimated correlation distributions, nonparametric. **Nonparametric smooth density estimates for selected ID pair subsets; for example, "DQ" labels the density estimate for the union of these disjoint groups from Figure 8: "D_Q only", "Affx &D_Q", "D_Q&EnV", and "all". The label "union" is all pairs regardless of which mapping resource was the source of the match.

Each ACC-probeset match is classified according to the set of annotation resources which returned the match. Figure 8 shows the distributions of these correlations, grouped by this classification. (The seven groups are mutually exclusive.) From the distributions seen in this figure, one confirms the widely reported fact that protein expression and mRNA expression often do not correlate strongly. However, there are differences among the 7 match groups. The nonparametric smooth density estimates of Figure 9 motivate the mixture characterization of the next section. The mixture model will accentuate the meaningful inter-group differences, which are between large positive correlations and all other correlations.

### Evaluation of mapping correctness by mixture modeling

The totality of observed Spearman correlations for all ID pairings were fitted to a two-component mixture distribution, where one density component was centered very near zero and the other had a positive mean. The posterior probability of membership in the second component was used as the target variable in regression analyses for evaluating the ability of each system to identify possibly correct matches. Using this posterior probability rather than the correlation itself focuses the effort of prediction on the part of the correlation distribution of interest.

One component, centered at 0.032 with standard deviation 0.124, has weight 66%. The other component, centered at 0.260 with standard deviation 0.189, has weight 34%.

**Figure 10:**
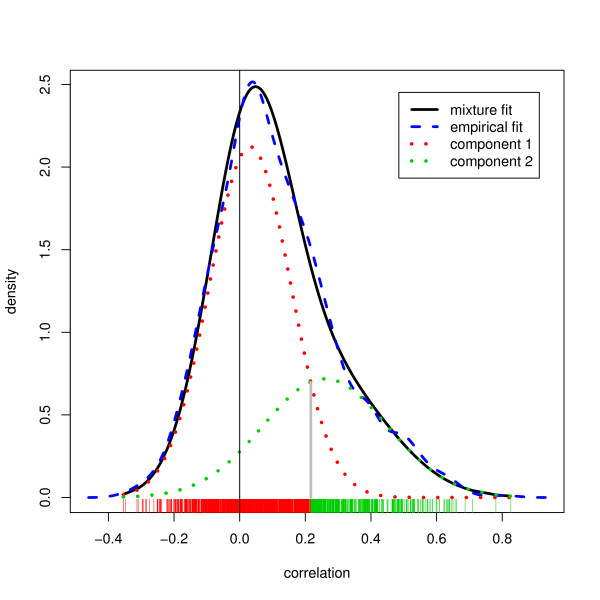
**Estimated correlation distributions, mixture model**. The correlation distribution smoothed, as a mixture. The black line is the estimated mixture distribution; the red and green are the estimated mixture components. For comparison, the orange line is an nonparametric smooth density estimate. The rug ticks are observed correlations, and the tick colors indicate group assignment by maximum posterior probability. Correlations greater than 0.217 have Pr(mixture component #2) > 0.5, and corresponding tick are colored green for component #2.

**Figure 11:**
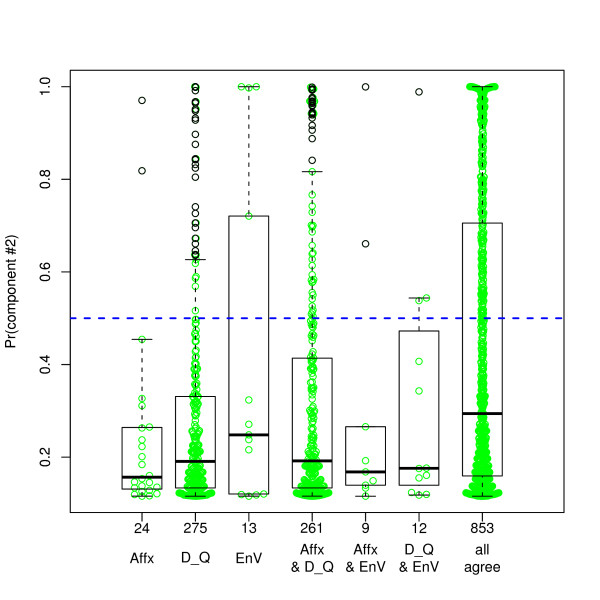
**Distributions of the "large correlation component" probability, for each match group**. Transformation of Figure 8, replacing the vertical correlation axis by the estimated probability of belonging to the second ("large correlation") component of the mixture shown in green in Figure 10. Horizontal line corresponds to a probability of 50%.

Figure 10 shows that the mixture model fits remarkably well. We do not claim that the correlations in reality come from a mixture distribution, though that is possible. Even if correct, membership in the first component may represent an incorrect match or a true biological disconnect between mRNA and protein abundance, and membership in the second component may or may not represent correct matches, since chance can generate extreme values. Nevertheless, the mixture model is extremely useful in this setting since the probability of membership in the second group, compared to using the correlation itself, is more sensitive to large correlations and relatively insensitive to differences between correlations that are not among the larger values. Therefore it makes a more useful dependent variable for the regression analyses to follow. The box plots of Figure 11 are similar to the box plots of Figure 8, but displaying the second component posterior probabilities rather than the correlations. The differences between match groups are considerably enhanced.

Linear regression analyses provided evaluations of the ability of each matching application to predict a high correlation. A linear regression models was fitted relating the presence or absence of a match in each mapping system to the component #2 probabilities.

The coefficient estimates from this model were: 0.119 for EnVision (P < 4 × 10^-10^), 0.039 for DAVID (P = 0.13), and 0.038 for NetAffx (P = 0.08). So, for example, if a match is returned by EnVision, the second component probability increased by 12.6% (= exp(0.119)-1). (Addition of total protein identification spectral count to the model did not affect the results. Here the weights were from the bootstrap analysis described in Methods. Similar results were returned when the normal theory weights were used.)

The results of this analysis suggest that a match in EnVision is more predictive of a good positive proteomic-genomic correlation, compared to matches provided by NetAffx or DAVID. However, this suggestion did not receive corroboration in head-to-head comparisons (example: pairs returned by EnVision but not NetAffx, compared to those returned by NetAffx but not by EnVision). Comparing pairs of disjoint groups from Figure 10, the one clear comparison, supported by large sample sizes, shows that a pair returned by DAVID and NetAffx is more likely to belong to the high-correlation cluster if it is also returned by EnVision (mean probabilities: 0.407 versus 0.290, P < 2 × 10^-7^). (We have used Spearman correlations throughout. Pearson correlations yield a somewhat higher second component probability, 48% instead of 34%, but shifted to lower correlation values within that component; overall the conclusions of the regressions are very similar.)

**Figure 12:**
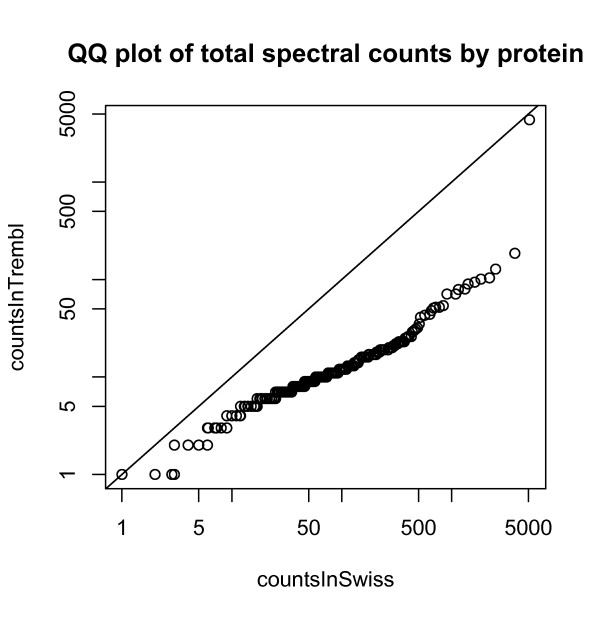
**QQ plot of total spectral counts by protein**.

**Figure 13:**
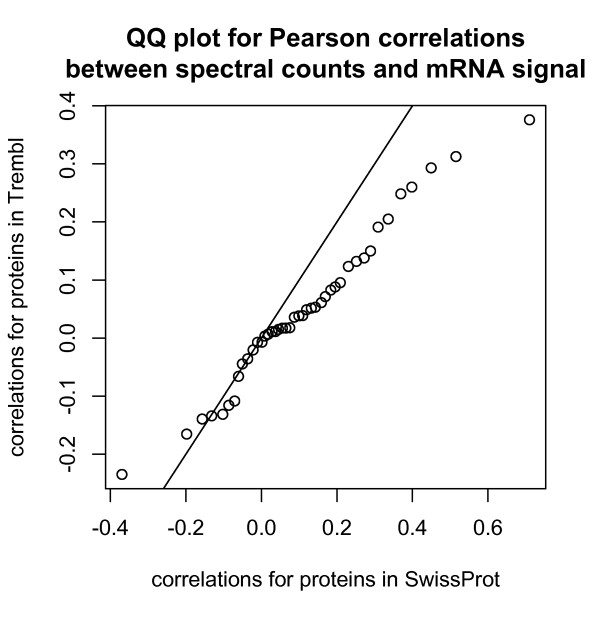
**QQ plot for Pearson correlations between spectral counts and mRNA signal,; restricting to 1573 pairs discussed above and shown in Figures 8, 9, 10, and 11**.

Filtering the ACCs further by restricting to SwissProt changed these results little; in fact this dropped only 3% (5 out of 480) ACCs, and 3% (43 out of 1573) of the ACC-probeset pairs. This reflects the fact that, over all ACCs, the association between SwissProt status and total spectral count is strong (Figure 12). SwissProt status is also associated with stronger correlations (Figure 13).

### Changes in the bioinformatics resources over time

In the past two years, there have been substantial changes in most of the services. The following table (table 6) shows the numbers of probeset mappings gained, lost, and maintained.

**Table 6:** Changes in probeset maps from proteomic experiment to U133 Plus 2.0.

	*DAVID_Q*	*DAVID_F*	*NetAff_Q*	*NetAff_F*	*EnVision_Q*
***added***	267	0	442	34	73
***dropped***	62	258	43	44	64
***maintained***	1134	1187	705	1113	814

A variety of analyses comparing added pairs to dropped pairs, or kept pairs to dropped pairs, revealed no evidence for NetAffx or for EnVision that the frequency of high correlations is changing. For DAVID_F, the ID pairs kept had significantly better correlations than those dropped (P = 2 × 10^-6^); similarly for DAVID_Q (P = 0.0001). However, the 267 pairs added recently in DAVID_Q were not superior to those dropped.

## Discussion

Integrating high-throughput biological data from multiple high-throughput platforms on the same set of samples will play an expanding role in basic biomedical research. Our picture of the molecular biology of tissue function now requires systems modeling. We would not understand the workings of an automobile if we only had access to a gas tank and a brake cylinder (or even 98 of each). We will be handicapped until we can pool perspectives on each and every important interacting molecular domain which we typically now view individually. This kind of integration of views from high-throughput platforms requires first a reliable mapping between the identifier lingua franca native to each platform. Bioinformatics resources to support this mapping are ever growing in number, comprehensiveness and sophistication. However, we have found that discrepancies between annotation resources mapping identifiers are surprisingly large. Uncritiqued erroneous mappings will lead to wasted effort in subsequent studies whose design and focus depended on the integrated study. On the other hand, when an interesting candidate molecule appears in one of the platforms, but ID mapping generates a poor correlation with the other platform, the integration can be useful by casting suspicion on the reported identity of the promising molecule. In the annexin 2 example, most but not all probesets mapping to annexin 2 yield good transcriptome/proteome correlations and a consistent view of differential expression.

We found that a match in EnVision predicts positive proteomic-genomic correlations the best, DAVID less so, and NetAffx the least. Nevertheless, as the boxplots of Figure 8 show, many matches found in DAVID and/or NetAffx but not in EnVision also had high correlations, and many matches reported by EnVision generated low correlations. Therefore, our results do not reduce to a clear recommendation to use one resource and not another. In fact, typical of many decisions, the trade-off between accepting a false match and rejecting a true match should govern the strategy.

The example presented here gives a concrete sense of the variability and discrepancy one can expect when attempting to integrate multiple-platform datasets by ID. Nevertheless, the primary value of this work is in the methodology, which has general utility for new integrative studies. The ID mapping resources studied here are evolving, as we have described. We deliberately restricted the focus of this study to bioinformatics resources providing direct mappings, but beyond this narrow scope one can conceive a multitude of indirect multiple-step strategies, using one or more intermediate identifiers, and possibly more than one resource[10]. Finally, new resources may well develop, providing new alternatives for ID mapping. Researchers engaging in integrated data analyses may well need to replicate the kind of study presented here, to compare their own selected mapping strategies on their own datasets. Therefore, the question of the best approach to linking these data will continue to be active and require dynamic answers. Furthermore, new platforms will expand the need for identifier mapping. Next generation sequencing will introduce new requirements, opportunities and challenges for multiple-platform data integration.

Several R packages are available through Bioconductor or directly from the authors (RSD), to conduct the analyses described herein on other pairs of identifier-indexed data sets. They include DAVIDQuery, EnvisionQuery, IdMappingRetrieval, and IdMappingAnalysis.

## Conclusion

Identifier mapping is a key step in integrative bioinformatics analysis. Evaluation of mapping services and strategies is both possible and valuable, despite the biological and technical causes that may lessen or destroy interplatform correlations. Integrative analysis may contribute by casting suspicion on molecule identifications. More positively, it may open doors to new biology by directing attention to a correctly mapped but decoupled interplatform pair of observations.

## Methods

### Tissue samples

Duke University Medical Center and the Gynecologic Oncology Group provided fresh frozen endometrial cancer tissue specimens from 91 stage I endometrial patients, under IRB-approved protocols at the corresponding institutions. Endometrial tissue specimens harvested by pathologists at the time of surgery were frozen until the time of the analysis. Hematoxylin and eosin (H&E) stained tissue specimens were evaluated by one of two board certified gynecologic pathologists to confirm the diagnosis. Seven age-matched normal endometrial samples from post-menopausal women comprised the control sample set.

Proteomic spectral count data on the same samples originated from Orbitrap and FT-ICR mass spectrometers. Details of the sample preparation, instrumentation, and spectral identifications are in Maxwell et al[14]. The gene expression data were from Affymetrix U133 Plus 2.0 microarrays. The raw data.cel and.chp files have been submitted to GEO in a MIAME compliant format (http://www.ncbi.nlm.nih.gov/geo/query/acc.cgi?token=bhmrrwmyyqsoeba&acc=GSE17025) and will be released July 8, 2011.

### Annotation systems

The identifier mapping systems examined included the Affymetrix NetAffx Analysis Center; ENFIN's EnVision and Ensembl resources; and the DAVID resource (Database for Annotation, Visualization, and Integrated Discovery by NIAID.

The NetAffx online resource[16], maintained by Affymetrix, permits the query of probe set information from Affymetrix GeneChip microarrays. A query takes as input a list of identifiers for genes, transcript or proteins, and provides (i) information on the development of each probe set including probe set target nucleotide sequence; (ii) corresponding gene, transcript and protein identifiers from public repositories; and (iii) connection information to other Affymetrix microarray experiments. The query provides immediate access to static information on the probe set and current public repository annotations. Below we refer to the mapping retrievals obtained interactively from NetAffx as **NetAffx_Q **or **Aff_Q**. Manually curated information is available as a CSV file which is updated on a quarterly basis. For the U133_Plus 2.0 chip, the file is specified as HG-U133_Plus_2.naXX.annot, where XX represents the current version iteration. The curation process is proprietary to Affymetrix, Inc.

The European Network of Excellence (ENFIN) )[17], a consortium for integrated systems biology, developed the EnCore integration platform. The EnCore Knowledge base connects relational data across multiple major bioinformatics databases including genome information (Ensembl), protein identification (PRIDE), pathway annotation (Reactome), molecular information (INAct) and gene Expression (ArrayExpress) to name a few. Two EnCore web graphical user interfaces (GUIs), EnVision and EnVision2, provide access to data for queries of limited size. EnCore also provides access to these bioinformatics resources through SOAP-based query web services with a common standard format, EnXML. Our study utilized the uniprot2affy service. Subsequent filtering restricted results to Human U133 Plus 2.0 Affymetrix probesets.

Ensembl is a joint project between European Bioinformatics Institute (EBI), an outstation of the European Molecular Biology Laboratory (EMBL), and the Wellcome Trust Sanger Institute (WTSI). The goal of Ensembl is to automatically annotate the genome, integrate this annotation with other available biological data, and make all this publicly available via the web. All Ensembl data can be accessed programmatically using the Perl API or through a GUI to retrieve unlimited amounts of data through bulk file download. Our study utilized the GUI to retrieve ID mapping data in the form of comma-separated-value (csv) file, filtered by species and microarray.

DAVID[11,22] is an annotation system developed by the National Institute of Allergy and Infectious Diseases at Frederick in conjunction with the Laboratory of Immunopathogenesis and Bioinformatics (LIB), SAIC Frederick. The DAVID Gene concept is defined for each known gene as a nonredundant annotation that unifies all functional annotation sources. The coverage of the DAVID Knowledgebase includes gene ontology and function, protein-protein interaction, disease association, literature references, protein domains and families, biological pathway, and gene expression annotation systems(12). DAVID offers a very simple DAVID Knowledgebase Query Tool API.

Matches to the UniProt set were obtained from the DAVID Knowledgebase using the DAVIDQuery R package available through the Bioconductor network[23]. This package utilized two functions in order to retrieve our representative dataset: *DavidQueryLoop*, which completes the query in one set of input rather than multiple submissions and *AffyProbesetList*, which restricts the UniProt to Affy probeset retrievals to only the Human U133 Plus 2.0 Affymetrix chip. In addition we obtained DAVID Knowledgebase files by request from the NIAID.

Results obtained variously in 2008 and 2009 are labeled as above but with the suffix "**_8**".

### Regression modeling

**Figure 14:**
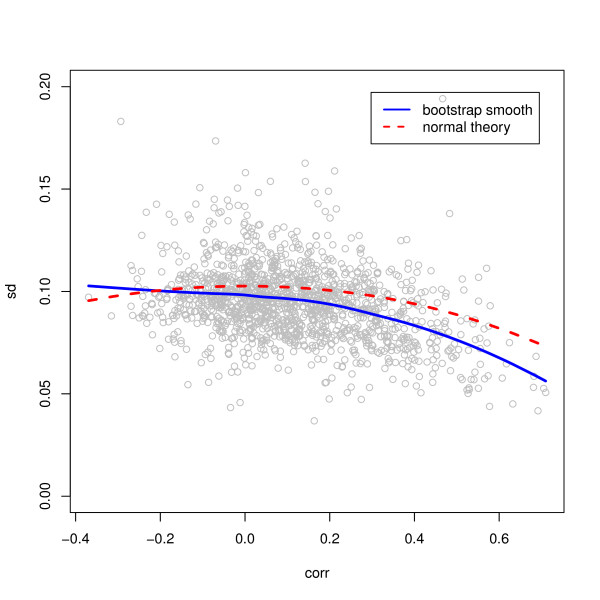
**Standard deviations of correlations. **Standard deviations of the correlations between transcript signals and spectral counts, by bootstrap with R = 200, with a nonparametric smooth, compared to the normal theory formula.

Regression weights were chosen as reciprocals of pair-specific variances, estimated in two ways: using the normal theory expression for the variance (1-ρ^2^)/(*n*-3) of a Pearson correlation coefficient estimate , and using a bootstrap variance estimate (R = 200 replications). The smooth fit for the relationship between the correlation and the bootstrap standard deviation follows the normal theory curve well except at large values, but the individual bootstrap estimates vary from the curve substantially (Figure 14).

## Authors' contributions

RSD performed primary coding and data analysis and drafted the manuscript. KKM performed data retrieval and data analysis. UC, TPC and GLM provided guidance, perspective, critique and editing. AL developed R packages to support the automated data retrieval and the data analysis, making the code efficient and widely applicable. TPC, BH, VSKK, and DK developed the proteomic data. TL and GLM developed the genomic data. All authors read and approved the final manuscript.
